# Genetic analysis of safflower domestication

**DOI:** 10.1186/1471-2229-14-43

**Published:** 2014-02-06

**Authors:** Stephanie A Pearl, John E Bowers, Sebastian Reyes-Chin-Wo, Richard W Michelmore, John M Burke

**Affiliations:** 1 Department of Plant Biology, Miller Plant Sciences, University of Georgia, Athens, GA 30602, USA; 2 The Genome Center, University of California, Davis, CA 95616, USA; 3 Center for Applied Genetic Technologies, University of Georgia, Athens, GA 30602, USA

**Keywords:** *Carthamus*, Domestication, Comparative genetic mapping, *Helianthus*, Parallel evolution, QTL analysis, Safflower, Sunflower

## Abstract

**Background:**

Safflower (*Carthamus tinctorius* L.) is an oilseed crop in the Compositae (a.k.a. Asteraceae) that is valued for its oils rich in unsaturated fatty acids. Here, we present an analysis of the genetic architecture of safflower domestication and compare our findings to those from sunflower (*Helianthus annuus* L.), an independently domesticated oilseed crop within the same family.

We mapped quantitative trait loci (QTL) underlying 24 domestication-related traits in progeny from a cross between safflower and its wild progenitor, *Carthamus palaestinus* Eig. Also, we compared QTL positions in safflower against those that have been previously identified in cultivated x wild sunflower crosses to identify instances of colocalization.

**Results:**

We mapped 61 QTL, the vast majority of which (59) exhibited minor or moderate phenotypic effects. The two large-effect QTL corresponded to one each for flower color and leaf spininess. A total of 14 safflower QTL colocalized with previously reported sunflower QTL for the same traits. Of these, QTL for three traits (days to flower, achene length, and number of selfed seed) had cultivar alleles that conferred effects in the same direction in both species.

**Conclusions:**

As has been observed in sunflower, and unlike many other crops, our results suggest that the genetics of safflower domestication is quite complex. Moreover, our comparative mapping results indicate that safflower and sunflower exhibit numerous instances of QTL colocalization, suggesting that parallel trait transitions during domestication may have been driven, at least in part, by parallel genotypic evolution at some of the same underlying genes.

## Background

The process of domestication, which has long been considered to be a form of “applied evolution,” inspired some of the earliest studies of evolution in response to natural selection [1]. Indeed, given the parallels between the adaptation of domesticated species to human-disturbed environments and the adaptation of wild populations to survival in natural environments [2], evolution under domestication is viewed by many as a valuable opportunity for studying the genetics of adaptation. Because many crop species share a common suite of traits (e.g., loss of seed dormancy, uniform flowering time, and fruit size) that evolved in response to selection during domestication (referred to as the “domestication syndrome”; [3]), comparative analyses across independent crop lineages also hold great promise for studying the genetic basis of parallel phenotypic evolution.

Over the years, quantitative trait locus (QTL) mapping has been used to investigate the genetic architecture of traits comprising the domestication syndrome in numerous crop species. Although early QTL-based studies suggested that domestication traits were predominantly controlled by a small number of large-effect QTL (e.g. [4-6]), other studies have revealed a higher level of genetic complexity (e.g. [7,8]). Comparisons among QTL analyses can also provide insight into the extent to which parallel phenotypic changes across independent crop lineages are driven by selection on homologous genes, or at least genomic regions. For example, comparative QTL mapping across crops in the Fabaceae [9], Poaceae [10,11], and Solanaceae [12] has provided evidence that many domestication traits, including increased seed weight, increased fruit size, and changes in flowering time and life history may be conditioned by independent changes in homologous genes in different lineages. Beyond providing fundamental evolutionary insights, such comparative genomic analyses also have the potential to aid in the improvement of other crops about which less is known. For example, knowledge that the *Arabidopsis* dwarfing gene, *GAI*, is structurally and functionally homologous to the wheat and maize dwarfing genes *RHT-B1, RHT-D1,* and *D8*, led to the transformation of the *GAI* gene into basmati rice to produce dwarf varieties [13].

In the present study, we investigate the genetic basis of the domestication syndrome in the oilseed crop safflower (*Carthamus tinctorius* L.; Carduoideae). Safflower is an annual, self-compatible, diploid (2*n* = 2*x* = 24; [14]) crop believed to have had a single origin of domestication in the Fertile Crescent region approximately 4000 years ago [15]. This species is well-adapted to growth in dry environments, having a long taproot (reported to grow over 1.5 m; [16,17]) that enables water uptake even when surface moisture is limiting. Originally, safflower was cultivated for its floral pigments (carthamine; [18]). Since its initial domestication, safflower cultivation has spread to other parts of the world, including many underdeveloped countries (e.g., Ethiopia, Afghanistan, and Sudan). Commercialization of safflower in the Americas began in the 1950s, where it has largely been used as an oilseed crop, in bird seed mixes, and as an ornamental species. Safflower is especially attractive as an oilseed crop, given that its seed oils are rich in mono- and polyunsaturated fatty acids. Though safflower possesses many of the standard traits that comprise the domestication syndrome (e.g., loss of seed dormancy, uniform flowering time, increased seed production, and increased seed oil quality and content), most cultivated safflower varieties have retained certain weed-like characteristics of their wild relatives (e.g., branching and leaf spines).

Safflower is a member of the Compositae (a.k.a. Asteraceae), which is currently recognized as the largest family of flowering plants [19,20]. This family contains ca. 10% of all flowering plant species [20] and includes over 40 economically important crops grown for a variety of uses, such as safflower, lettuce (*Lactuca sativa* L.; Cichorioideae), and sunflower (*Helianthus annuus* L.; Asteroideae). These three crops represent the three major subfamilies within the Compositae, which collectively account for 95% of the species diversity within the family. Like safflower, sunflower is primarily grown as an oilseed crop. Given this, along with the wealth of available information on the origin and evolution of cultivated sunflower (e.g. [7,8,21-25]), our work also provides an opportunity to study the genetic basis of parallel phenotypic changes during domestication within this important family.

Here, we describe a genetic map-based study of domestication-related traits in a population derived from a cross between safflower and its wild progenitor (*C. palaestinus* Eig.; see below). Our results indicate that the genetic architecture of safflower domestication is complex, with the majority of traits being controlled by multiple QTL with small to moderate phenotypic effects. Moreover, a comparison of our results to those derived from similar analyses in sunflower provides evidence of QTL colocalization, highlighting possible parallels in genetic architecture between safflower and sunflower and, in some cases, suggesting that parallel trait transitions may have been driven by parallel genotypic changes in these lineages.

## Methods

### Mapping population

Seeds obtained from the USDA for both safflower (cv AC Sunset; PI 592391) and *C. palaestinus* (PI 235663) were germinated in the University of Georgia greenhouses during the summer of 2009. AC Sunset is an inbred, dual-purpose (i.e., birdseed and oilseed) cultivar developed in Canada [26]. Like many other high oil varieties, the leaf margins of AC Sunset plants have prominent spines. Genetic analyses based on nuclear and chloroplast markers [27] as well as archaeological [28] and geographic evidence [18,29] all point to the predominantly selfing *C. palaestinus* (2*n* = 2*x* = 24; [30]) as the wild progenitor of safflower. This species is native to the Middle East in the area around Israel and is fully cross-compatible with safflower. Though it exhibits considerable morphological variation for a variety of traits including leaf spininess, leaf shape, duration of rosette habit, and flower color, *C. palaestinus* can be distinguished from safflower based on its tendency toward non-uniform germination, an extended rosette habit, and smaller seed size. Also, contrary to the expectation based on most crop-wild comparisons, *C. palaestinus* exhibits more limited branching than safflower ([31]; unpublished observation).

A single safflower plant served as a pollen donor in a cross between safflower and its wild progenitor. The F_1_ seeds from this cross were germinated and the resultant plants were selfed to produce F_2_ families, the largest of which was chosen for use in the QTL analysis described herein. A mapping population consisting of 276 F_2_ individuals was grown and phenotyped in the greenhouse during the summer of 2010. Additionally, nine plants of the inbred AC Sunset and nine selfed offspring of the *C. palaestinus* mapping parent were grown in the greenhouse alongside the mapping population to provide estimates of parental trait means under the same conditions as the mapping population. The mapping population and parental plants were transplanted into 12-inch tall treepots (Stuewe and Sons, Tangent, OR) and grown in a completely randomized fashion within a single greenhouse room. All pots were moved weekly throughout the duration of the study to minimize the effects of micro-environmental variations within the greenhouse.

### Phenotyping

Plants were checked daily and dates were recorded for estimates of root growth rate and the initiation of flowering. Root growth rate was based on the number of days until the roots reached the bottom of each pot. Leaf size, shape, perimeter, and spininess were estimated and averaged across three leaves collected from each plant (the most recent fully expanded leaf, the leaf directly below the primary capitulum, and the longest rosette leaf) and scanned for analysis with ImageJ v1.43u [32]). Spininess was measured using a modified version of the spine index, which was initially described in [33] as the number of spines on a leaf multiplied by the length of the longest spine on that leaf. Here, a “standardized spine index” was used, taken as a measure of the number of spines per centimeter of leaf margin multiplied by the length of the longest spine on that leaf.

Heads were bagged on the day of anthesis to prevent cross-pollination and potential seed loss. The height and diameter of the primary head and disc were measured using digital calipers on the day of first flowering of each plant. Stem height was measured as the length of the stem from the base of the plant to the base of the primary head. Fresh florets were collected from the third flowering head on the day that it opened and mature florets were collected from the primary head after all flowering had ceased. These florets were flash frozen in liquid nitrogen to preserve their pigments, which were later measured with a Gardner XL20 colorimeter (Bethesda, MD) using coordinates from CIE L*a*b* color space (in which L* represents luminosity and a* and b* represent the coordinates of each pigment’s hue on the red/green and blue/yellow axes, respectively; [34,35]). For each measurement, differences in hue due to differences in light intensity were controlled for by holding L* constant at 30 units. Because floret color changes from yellow (anthesis) to red (senescence) in AC Sunset (but not in the wild progenitor), we recorded the magnitude of floret color change in each plant by calculating the difference between the a* value at flowering and the a* value at maturity. Smaller a* values correspond to yellower flowers and larger values correspond to redder flowers, and b* values changed marginally between these two colors.

Heads were harvested at physiological maturity (i.e., when the bracts were no longer green). Seven days after harvest, 12-16 achenes were sterilized using a 10% bleach solution and planted at a 1.5 cm depth into small pots maintained in a growth chamber for the assessment of seed dormancy and viability. Plants with primary heads lacking sufficient seed set (n = 119) were omitted from this analysis. Pots were monitored daily for up to 60 days and dates of seedling emergence were recorded and used to estimate the fraction of achenes that germinated within the 60 day window and calculate the average time to germination for each F_3_ family.

At the conclusion of flowering, the number of senesced heads, the height (above the soil) of the lowest branching point, the number of internodes, and the length of the second internode were recorded for each plant. As the remaining heads reached maturity, selfed achenes were harvested, counted, weighed, and measured. Average achene weight was based on a random subset of 50 achenes. However, for plants that produced less than 50 achenes, the average achene weight was based on all achenes produced.

Seed oil content and composition were estimated for all plants with sufficient seed set. These measurements were taken following previously established protocols (percent oil content: [21,36]; seed oil composition: [24]). Briefly, a Bruker MQ20 minispec NMR analyzer (The Woodlands, TX) was used to determine percent oil content. The standard protocol was modified to accommodate measurements based on small seed sets by placing ca. one cm of tissue paper into the flat-bottomed tubes and adding ca. one cm of cleaned safflower seeds on top of the tissue paper. Percent oil content was estimated using a calibration curve using commercial safflower oil as a standard (Hollywood, Boulder, CO). Oil composition was determined based on gas chromatography of fatty acid methyl esters. A total of ten achenes from each plant were hand ground and fatty acids were extracted and then analyzed using an Agilent 6890 N gas chromatograph (Santa Clara, CA).

### SNP identification

Single nucleotide polymorphisms (SNPs) that differentiated the parents of our population were identified from expressed sequence tag (EST) and transcriptome data generated from each parent. For the cultivated mapping parent, we used the AC Sunset EST data produced via Sanger sequencing of cDNAs described in Chapman et al. [37]. For the wild mapping parent, we produced transcriptome sequence data via 454 sequencing (454 Life Sciences, Branford, CT) as follows: RNA was extracted from mature leaves, bracts, florets, and developing ovules collected from a single *C. palaestinus* plant using a combined trizol (Invitrogen, Carlsbad, CA) and RNeasy mini column (Qiagen, Valencia, CA) method. RNA extracted from each tissue type was pooled in equal proportions, normalized prior to 454 library preparation following the protocols described by Lai et al. [38] and Meyer et al. [39], sequenced, and assembled using MIRA v3.0.3 [40] (see Additional file 1).

The assembled sequences from the mapping parent assemblies were aligned to each other using Mosaik [41]. SNPs were identified using SAMtools [42] and run through the Illumina GoldenGate “assay design tool” (San Diego, CA; http://illumina.com/), which identified SNPs free of other polymorphisms within 60 bp of the targeted SNP site and assigned a quality score predicting the success with which a SNP would be assayed. To facilitate downstream comparative genomic analyses, SNPs used in this study were preferentially selected from *Carthamus* unigenes with mapped homologs in the high-density sunflower “consensus” map [43]. A total of 384 SNPs meeting design requirements were targeted for genotyping, and a subset of these were validated via genetic mapping (see below).

### Genotyping and map construction

Whole genomic DNA was extracted from leaf tissue from each F_2_ plant as well as the mapping parents using a modified CTAB protocol [44]. DNA concentrations were estimated using the Quant-iT PicoGreen kit (Invitrogen) using a Biotek Synergy 2 plate reader (Winooski, VT). The Illumina GoldenGate Assay described above was then used to genotype each sample on the BeadXpress Reader (Illumina) at the Georgia Genomics Facility. Allele calls were obtained using the Illumina GenomeStudio software v2011.1.

A genetic linkage map was constructed using MapMaker 3.0/EXP [45,46]. Briefly, initial linkage groups (LGs) were identified using the “group” command with a minimum LOD score of 5.0 and a maximum frequency of recombination of 0.4 between adjacent markers. Preliminary map orders were determined using the “compare” command on a subset of markers within LGs and the remaining markers were placed using the “try” command. For each LG, marker orders were confirmed using the “ripple” command and the final marker orders, presented here, represent the most likely marker orders given the data.

### Statistical analyses and QTL mapping

Histograms and trait means of the mapping population and mapping parents were plotted for visualization using the R Statistical Package [47]. Estimated parental trait values were further analyzed to test for significant differences. This was done using either Welch’s *t-*tests or, when trait distributions deviated significantly from normality (as determined by the Shapiro-Wilk test for normality), Wilcoxon signed-rank tests. Spearman correlation coefficients were calculated among all traits measured in the F_2_ mapping population using the “hmisc” package [48] in R [47]. Significance was determined using the sequential Bonferroni method with α = 0.05 [49].

QTL were identified using QTL Cartographer v1.17j [50,51] following established approaches (e.g. [7,8,21,24]). Briefly, composite interval mapping (CIM) was performed in ZmapQTL with a 10 cM window and a maximum of five background cofactors identified using SRmapQTL with forward/backward stepwise regression, and tests were performed at 2 cM intervals. Permutation thresholds (*α* = 0.05 and 0.1) for declaring QTL significance were estimated based on 1000 permutations for each trait [52,53]. Secondary peaks were not considered as separate QTL unless there was a 2-LOD decline between adjacent peaks.

The results generated from CIM were then used as an initial model for multiple interval mapping (MIM), as implemented by MIMapQTL [54]. This analysis was used to confirm QTL identified via CIM. Following the authors’ recommendations, the information criterion was set as IC(k) = -2(log(L)-kc(n)/2), where c(n) = log(n) was the penalty function and the threshold was set at 0. Epistasis was investigated at a genome-wide level using EPISTACY v2 [55] to test for interactions between all possible pairs of codominant markers that exhibited unique segregation patterns (i.e., redundant markers that showed identical segregation patterns were joined into a single haplotype to reduce the number of pairwise comparisons). As suggested by the author, significance was determined by dividing the comparison-wise error rate (*α* = 0.05) by *g*(*g*-1)/2, where *g* is the haploid number of LGs in safflower (n = 12).

The mode of gene action for each QTL was estimated by dividing the dominance effect of the cultivar allele by its additive effect (d/a), such that cultivar alleles that are completely recessive have a value of -1 and those completely dominant have a value of +1. Following the cutoffs employed by Burke et al. [7], the mode of gene action of the cultivar allele at each locus was categorized as follows: underdominant ≤ -1.25 < recessive ≤ -0.75 < partially recessive ≤ -0.25 < additive < 0.25 ≤ partially dominant < 0.75 ≤ dominant < 1.25 ≤ overdominant. Additionally, the magnitude of the effect of each QTL was considered to be “large” if the percentage of segregating phenotypic variance explained (PVE) was greater than 25%, “small” if the PVE was less than 10%, and “intermediate” if in between these values.

### Comparative genomic analyses

In order to identify homologous loci between the safflower and sunflower genomes, all ESTs harboring safflower SNPs mapped in this study as well as all loci from the 10,000 feature sunflower consensus map [43] were compared via BLAST to the lettuce genome, v4 [56]. As noted above, lettuce is a member of the Cichorioideae, which falls at an intermediate phylogenetic position between the Carduoideae and the Asteroideae. The use of the sequenced lettuce genome as an intermediary greatly simplified these analyses because it is the same ploidy level as safflower (though functionally diploid, sunflower is a paleopolyploid [i.e., tetraploid relative to safflower and lettuce] due to an ancient whole genome duplication at the base of the Heliantheae; [57]) and because it dramatically increased the number of mapped loci bridging the safflower and sunflower maps (i.e., virtually all of the ESTs from which the safflower and sunflower markers were derived could be matched to corresponding sequences in the lettuce genome). The top two BLASTN hits with an e-value better than 1x10^-6^ were recorded and, to facilitate comparative QTL mapping, efforts were focused on establishing homology across genomes in regions of the sunflower genome containing relevant QTL (see below). Pairs of linkage groups across species that had three or more homologous loci (as determined by BLAST) were considered to be putatively homologous (i.e., syntenic) chromosomal regions. Instances where multiple chromosomal regions containing relevant sunflower QTL exhibited homology to one or more regions in lettuce, perhaps due to the duplication history of these species, were retained for further analysis, as were cases in which multiple lettuce LGs exhibited homology to one or more safflower LGs. Finally, QTL-containing regions exhibiting 1:1:1 homology across the three genomes were also retained.

We then surveyed the literature to identify previously mapped sunflower domestication QTL for traits homologous to those investigated in this study (Additional file 2). Because many of the markers used to map these QTL were included in the sunflower consensus map [43], it was possible to project the positions of these QTL onto that map for comparative QTL mapping. For instances in which the bounds of 1-LOD intervals could not be directly projected onto the consensus map (due to an absence of shared markers at the 1-LOD boundaries), we estimated the distance from shared markers within the 1-LOD interval to the boundaries based on relative map lengths. To determine the probability that instances of QTL colocalization were due to chance alone, we used the hypergeometric probability distribution function (‘sampling without replacement’; [58]); as described in Paterson et al. [11] and Paterson [59] as follows:

$$p=\frac{\left({}_{m}^{l}\right)\left({}_{s-m}^{n-l}\right)}{\left({}_{s}^{n}\right)}$$

where *n* is the number of intervals that can be compared (estimated here as the genome size divided by average QTL size for a given trait), *m* is the number of colocalizing QTL, *l* is the total number of QTL in the larger sample, and *s* is the number of QTL in the smaller sample for a given trait.

## Results

### Phenotypic analyses

**Figure 1:**
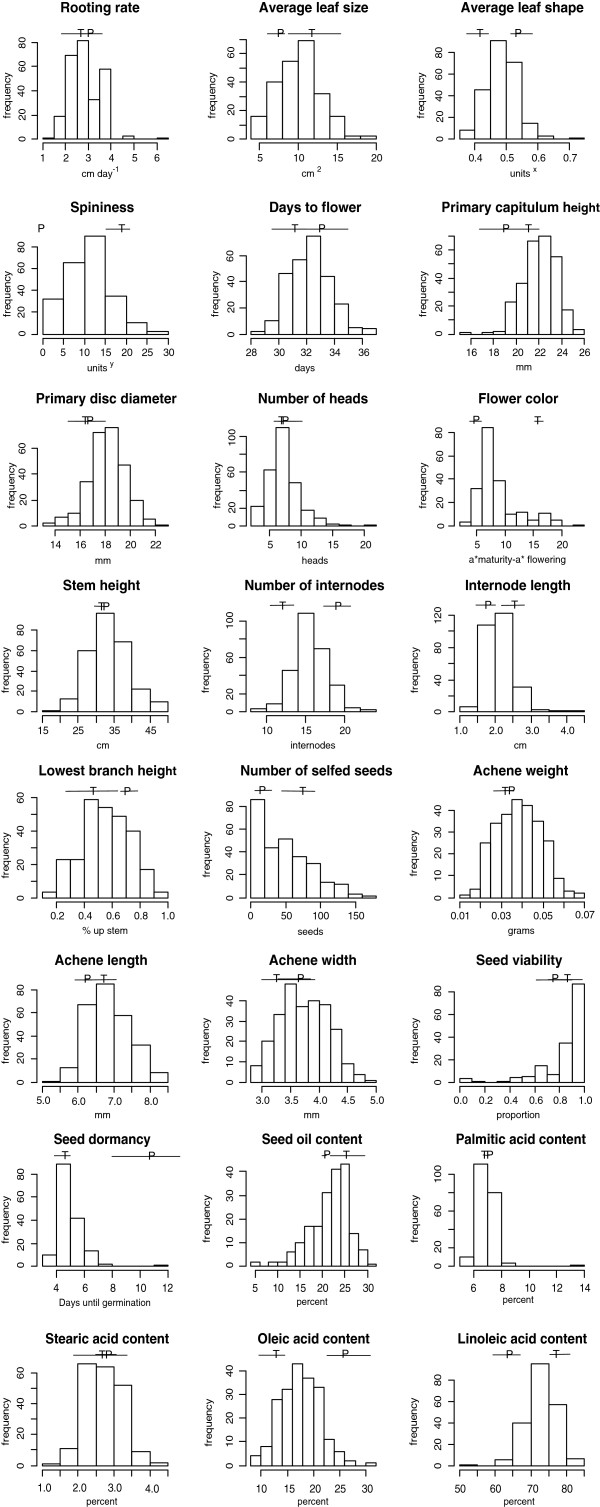
**Trait distributions of the F**_**2 **_**mapping population and mapping parent representatives.** Means of the mapping parent representatives are represented by a T (*C. tinctorius*) and P (*C. palaestinus*) and solid lines represent one standard deviation around the means.

Of the 24 traits analyzed, 15 differed significantly between the mapping parents when grown alongside the mapping population (Table 1). Comparisons of the means and standard deviations of the mapping parent representatives to the F_2_ trait distributions revealed transgressive segregation for the majority of the traits analyzed (i.e., there are F_2_ individuals with trait values exceeding one standard deviation in either direction of the mapping parents; Figure 1). The most extreme examples of transgressive segregation were for traits related to vegetative growth: capitulum height, disc diameter, stem height, leaf size, and achene size. In other words, many of the F_2_ plants and their achenes were larger than either of the mapping parents.

**Table 1:** Average trait values of mapping parents

**Trait**	***Carthamus palaestinus *** **(progenitor)**	***Carthamus tinctorius *** **(cultivated safflower)**
Rooting rate (cm day^-1^)	**3.24**	**2.41**
Average leaf size (cm^2^)	**7.352**	**12.3**
^x^Average leaf roundness	**0.559**	**0.412**
Spininess (^y^spine index/leaf perimeter)	**0**	**19.031**
Days to flower	**33.29**	**31**
Primary capitulum height (mm)	**18.97**	**21.13**
Primary disc diameter (mm)	16.94	16.76
Number of heads	8.78	8.63
Flower color (lab color space a* units)	**5.47**	**16.88**
Stem height (cm)	32.94	31.36
Number of internodes	**19.11**	**12.33**
Internode length (cm)	**1.74**	**2.56**
Lowest branch height (percent up stem)	**72**	**47**
Number of selfed seed	**12.44**	**68.59**
Achene weight (mg)	33.8	31.2
Achene length (mm)	**6.23**	**6.63**
Achene width (mm)	**3.63**	**3.38**
Seed viability (percent)	75	84.9
Seed dormancy (average number of days until germination)	**10.95**	**4.31**
Seed oil (percent)	**21.35**	**26.29**
Palmitic acid (percent)	6.97	6.78
Stearic acid (percent)	2.79	2.57
Oleic acid (percent)	26.69	12.72
Linoleic acid (percent)	63.55	77.93

**Bold** indicates trait values that are significantly different from one another (t-test, p < 0.05).

^x^Average leaf roundness = 4 × [leaf area/(π × (major leaf axis)^2^], where values closer to 1 represent circular shapes and values closer to 0 represent oblong shapes.

^y^spine index = number of spines × length of longest leaf spine (in mm).

Approximately one-fourth of the mapping population had florets that developed a deep red color at maturity. The flower color ratio did not differ significantly from a 3:1 distribution (χ^2^ = 0.16, *P* = 0.6; data not shown). This suggests that flower color variation in the mapping population is controlled by a single gene, with the ability to turn red being recessive.

Many of the traits under study were correlated within the F_2_ mapping population (Additional file 3) in a way that was largely consistent with the observed parental trait combinations. Several of these correlations, however, were no longer significant after Bonferonni correction. Notably, the total number of selfed achenes produced was positively correlated with achene oil content (ρ = 0.573, *P* < 0.001) and stem height (ρ = 0.420, *P* < 0.001) and negatively correlated with achene dimensions (achene weight: ρ = -0.562, *P* < 0.001; achene width: ρ = -0.558, *P* < 0.001; achene length: ρ = - 0.730, *P* < 0.001).

### Genetic mapping

**Figure 2:**
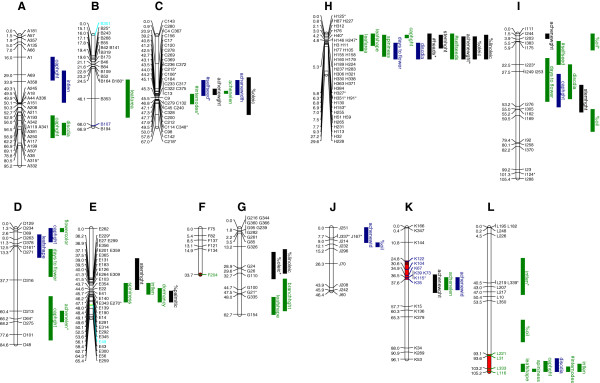
**Genetic map of the safflower genome and corresponding quantitative trait locus (QTL) positions.** Marker names are listed on the right and positions (in cM) are listed on the left of each linkage group. Markers with an asterisk represent SNPs mapped as dominant markers. Bars represent 1-LOD QTL intervals and traits with an asterisk denote “low confidence” QTL (α = 0.10). Green bars show locations of QTL in which the cultivar allele exhibits phenotypic effects in the expected direction while dark blue bars represent QTL where the cultivar allele confers a wild-like phenotype. Black bars represent QTL for traits in which the parents did not exhibit significant differences. Shaded regions along each linkage group represent regions exhibiting segregation distortion, with the following colors indicating different significance levels: yellow α < 0.05, brown α < 0.01, and red α < 0.001. Marker names are shaded the same colors as QTL bars to indicate the directionality of the distortion, where light blue indicates that there is a heterozygote excess.

Of the 384 SNPs designed for the Illumina GoldenGate assay, 244 exhibited interpretable polymorphisms that could be used for genetic mapping (Additional file 4). The remaining 140 markers were omitted due to monomorphism in the mapping population, overly complex segregation patterns (presumably due to paralogy), or failure of the assay probes to hybridize with the target DNA. Although SNPs are typically scored as codominant markers, 26 of the SNPs included in this study were scored as dominant markers due to the lack of a clear distinction between one of the homozygote classes and the heterozygote class (these markers are flagged with an asterisk in Figure 2).

The 244 markers coalesced into 12 LGs, consistent with the haploid number of n = 12 chromosomes that has previously been reported for safflower (Figure 2; [30]). These LGs ranged in size from 30.7 to 105.3 cM (average = 71.5), with each group containing 6 to 40 markers (average = 20.3). A Poisson goodness-of-fit test revealed that the mapped markers were non-randomly distributed along the linkage groups (*P* < 0.0001). The total map length summed to 858.2 cM, resulting in an average intermarker distance of 3.7 cM (range = 0.0-39.6 cM).

The segregation patterns of 14 out of the 244 mapped markers deviated from Mendelian expectations (i.e., they exhibited significant segregation distortion after Bonferroni correction; [60-62]. Eleven of these loci were located in two distorted regions spanning 13 cM (on LG K) and 12 cM (on LG L; Figure 2). Distortion occurred in both directions: in some cases, the wild allele was overrepresented in the mapping population while in other cases, the cultivar allele was overrepresented. Two markers exhibited an underrepresentation of both homozygote classes, yielding a heterozygote excess. Within each of the two aforementioned distorted regions, however, the direction of deviation remained consistent. The segregation of the markers on LG K was consistently skewed in favor of the wild allele, whereas the segregation of the markers on LG L was consistently skewed in favor of the cultivar allele. Further, the magnitude of the distortion was significantly greater on LG L (*P* = 0.01).

### QTL mapping

A total of 61 QTL were identified for 21 of the 24 traits studied (Table 2, Figure 2). Only LG F lacked QTL. Eight of the sixty-one QTL were marginally significant, having been identified at the *α* = 0.1 permutation threshold during CIM; the remainder exceeded the *α* = 0.05 permutation threshold and all of these QTL were confirmed via MIM. The 1-LOD confidence intervals for these QTL averaged 13.5 cM, ranging from 1.5 to 31.9 cM. Of the 21 traits, 16 traits had multiple QTL (range = 2-7). Nearly all mapped QTL 1-LOD intervals overlapped at least 1 of the 244 mapped markers, with the lone exception of a QTL for% oil that mapped between markers on LG L. One trait had two instances of antagonistic QTL on the same LG (capitulum height; on both LGs A and D), where the cultivar-derived allele for one QTL conferred a cultivar-like phenotype and the cultivar-derived allele for the other QTL conferred a wild-like phenotype. The majority of all QTL identified mapped to one of seven QTL clusters on seven different chromosomes, with each cluster harboring three to twelve QTL, and a Poisson goodness-of-fit test revealed that the observed QTL were non-randomly distributed (*P* < 0.0001). Also, 23 of the QTL identified in this study mapped to 3 of 7 genomic regions that exhibited marker clustering (LGs C, E, and H), and a bionomial goodness-of-fit test (*P* < 0.0001) indicates that the distribution of markers and QTL are significantly non-independent.

**Table 2:** Quantitative trait locus (QTL) positions, modes of gene action, and magnitudes of effect for 19 out of the 24 traits studied

**Trait**	**Linkage group**	**Position**^**a**^	**Nearest marker**	**1-LOD interval**^**b**^	**Additive effect**^**c, d**^	**Dominance ratio**	**PVE**
Average leaf size	B	52.1	B353	32.6-60.1	0.44	-3.69	8.7
	H	8.6	H113	0.9-12.5	1.26	0.69	9.9
Average leaf roundness	D	10	D378	4.6-21.3	**0.02**	-0.05	10.4
	G	56.3	G154	50.3-62.3	-0.02	-0.36	13.1
	H	4.3	H40	0-8.6	-0.02	-0.37	7.7
	L	105.3	L116	99.7-105.3	-0.02	0.82	4.5
Spininess	E	48.2	E190	40.1-53.9	1.74	0.13	4.5
	H	5.4-6.1	H327	0.9-16.5	2.93	-0.21	14.4
	L	105.3	L116	103.3-105.3	5.92	0.65	32.7
Days to flower	D	25.3	D271	15.3-35.3	-0.73	0.06	11.9
	H	8.8	H113	6.7-18.5	**0.42**	1.25	5.6
	I	35.5	I253	17.9-49.5	-0.49	-0.58	6.4
Primary capitulum height	A	24	A69	16.0-33.0	**-0.67**	0	8.7
	A	62.2	A117	58.8-75.0	0.39	-0.8	4.5
	D	0.01	D129	0.0-10.0	**-0.61**	-0.23	8.7
	D	68.3	D275	49.7-81.6	0.43	0.11	4.5
	H	2.1	H312	0.9-3	0.66	0.57	9.5
	I	41.5	I276	29.5-53.5	**-0.64**	-0.14	9.9
	L	101.7	L333	97.7-105.3	0.66	1.08	6.1
Primary disc diameter	A	66.8	A199	64.2-73.0	-0.60	-0.46	9.2
	H	12.5	H113	6.7-18.5	**0.76**	0.25	12.3
	I	35.5	I253	22.5-47.5	-0.60	0.18	9.6
	L	101.7	L333	95.7-105.3	**0.72**	0.15	8.2
Number of heads	H	3*	H76	0.0-18.5	*-0.56*	-1.4	4.8
Flower color	D	1.3	D234	0.0-2.6	3.70	-0.79	63.4
Stem height	E	37.2	E201, E359	22.0-44.8	*2.18*	-0.01	6.9
	H	6.7	H130	0.9-10.5	*2.31*	0.42	7.8
	I	53.2	I276	37.5-57.2	*-1.88*	0.52	6.3
Number of internodes	C	47.5*	C200	43.1-50.3	-0.63	-0.44	4.4
	L	105.3	L116	101.7-105.3	-1.46	0.07	15.9
Internode length	A	41.8	A245	26.0-49.2	**-0.14**	0.43	7.6
	E	43.8	E354	40.1-48.2	0.13	0.32	4.6
	L	40.5*	L219, L339	22.5-49	0.10	0.86	4.2
	L	105.3	L116	99.7-105.3	0.17	0.1	6.7
Lowest branch height	G	44.9	G100	36.9-60.3	-0.06	-0.22	5.9
Number of selfed seed	C	42.6*	C278	33.0-44.2	**-2.17**	7.26	4.2
	H	7.3	H255	0.0-18.5	15.89	-0.14	7.6
	I	13.9	I175	5.5-22.5	13.71	0.29	6.9
Achene weight	C	42.6	C278	39.3-43.5	*-0.33*	-21.68	11
	H	7.8*	H231	2.1-18.5	*1.71*	2.51	4.4
	I	0.0	I111	0-3.5	*-4.96*	0.73	13.1
	K	37.6	K35	34.8-47.6	*4.67*	0.06	8.2
Achene length	C	41.3	C120	40.9-42.7	0.08	5.004	10.4
	D	62.4*	D213	60.4-68.3	0.15	0.813	5.2
	I	17.9	I223	5.9-21.9	**-0.28**	0.155	12.0
	K	37.6	K35	36.5-45.6	0.29	-0.349	8.2
Achene width	C	42.6	C278	33.0-43.5	**0.09**	2.69	8.6
	I	0.0	I111	0.0-1.5	-0.21	0.8	15.3
	J	11.1	J232	0.0-11.1	**0.13**	0.37	5.1
	K	37.6	K35	36.5-45.6	**0.18**	-0.11	6.8
Seed dormancy	E	48.6	E190	43.8-53.9	-0.47	0.4	9.0
Seed oil	I	3.9*	I203	0.0-11.9	1.61	0.81	6.4
	I	65.4	I92	55.2-71.4	1.67	1.04	10.6
	J	12.2	J232	11.1-14.2	**-1.73**	0.21	7.2
	L	75.6	L221	67.6-83.6	2.65	1.35	23.2
Palmitic acid	E	46.4	E140	45.8-53.9	*0.27*	0.6	7.5
Oleic acid	C	50.3	C98	31.0-58.1	*-1.33*	-0.34	6.4
	G	29.8*	G26	17.4-37.0	*1.28*	0.71	6.3
	H	5.4-6.1	H327	2.1-18.5	*-1.55*	-0.66	11.0
Linoleic acid	G	31.8	G110	15.4-32.9	*-1.69*	0.67	8.6
	H	5.4-6.1	H327	0.9-18.5	*1.47*	-0.78	8.7

^a^Absolute position from the top of the linkage group, in cM.

^b^Region flanking the QTL peak within a one LOD score decline of the peak.

^c^Refers to the effect of the cultivated safflower allele.

^d^**Bold** values indicate QTL in the “wrong” direction (see text for details) while italicized values describe cases in which directionality cannot be determined due to similarity in average parent trait values.

*Describes lower confidence QTL, identified at α = 0.1

The PVE for the identified QTL ranged from 4.2% to 63.4% (Table 2), with the majority of the QTL having small effects (PVE <10%). There were 13 QTL with intermediate effects and just 2 QTL with large effects (spininess and flower color, 32.7% and 63.4% respectively). For traits that differed significantly between the mapping parents, it was possible to investigate whether the respective QTL had allelic effects in the expected direction (i.e., whether or not the cultivar allele produced a more cultivar-like phenotype). Examination of the 44 QTL identified for the 15 traits with means differing significantly in the parents revealed 12 QTL for 9 traits conferring phenotypes in the “wrong” direction. For the remaining three traits for which multiple QTL were identified, the effects of all QTL were consistent with the observed parental trait differences, even if those differences were not significant (average leaf size, spininess, and number of internodes; Table 2). Interestingly, the majority of the seed width QTL had cultivar alleles conferring a wild-like phenotype. The mode of gene action of each QTL ranged from -21.7 (average seed weight) to 7.3 (total number of seeds per plant), though the majority fell in the -1 to 1.25 range (average ± S.E. = 0.72 ± 0.40). Traits with overdominant QTL included flowering time, total number of seeds, seed weight, seed length, seed width, and seed oil content; those with underdominant QTL included leaf size, number of capitula, and seed weight.

The genome-wide scan for epistasis detected a total of 105 significant epistatic interactions at the α < 0.05 level (after correcting for multiple comparisons) for 19 traits (Additional file 5). Note that multiple interactions detected among loci on the same linkage groups were counted as a single interaction, accounting for the non-independence of these loci due to linkage. Not all traits with QTL were found to be influenced by epistatic loci and some traits only appeared to be influenced by loci with epistatic effects. On average, 5.5 ± 1.27 interactions were identified for the traits with epistasis, ranging from 1 to 19 significant interactions per trait. Additive-by-additive and additive-by-dominant interactions comprised the majority of the interactions (40 and 42 interactions, respectively). Upon closer inspection of the EPISTACY results, we noticed two traits that had QTL × QTL interactions: leaf shape (LG H × L, additive-by-additive) and seed length (LG K × C, additive-by-dominant and dominant-by-dominant).

### QTL colocalization

**Figure 3:**
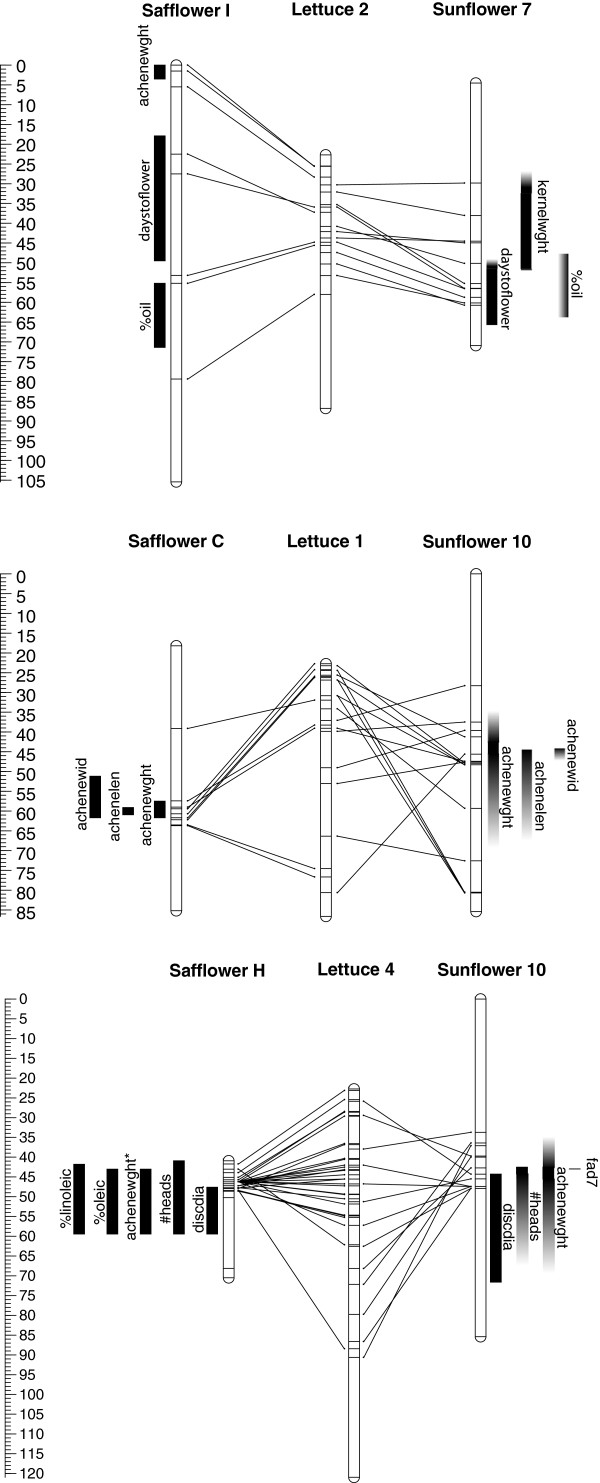
**Comparative mapping of the safflower, lettuce, and sunflower genomes.** Lines connect homologous loci between genomes. Black bars indicate quantitative trait loci (QTL) with exact 1-LOD positions known, while grayed gradient sections of bars represent estimated positions of QTL (based on relative lengths of the Bowers et al. [43] consensus map and the maps in which the sunflower QTL were originally published). Traits with an asterisk denote “low confidence” safflower QTL significant at α = 0.1.

We were somewhat limited in our ability to directly identify instances of QTL colocalization between safflower and sunflower due to the relative paucity of markers in safflower and, consequently, a limited number of shared markers between the maps. The lettuce genome sequence, however, served as an effective intermediary in bridging the gaps between these maps. We ultimately identified 14 QTL corresponding to 10 different domestication-related traits in safflower (1 to 3 QTL per trait) that colocalized with previously identified QTL in sunflower (Figure 3, Additional file 6; [7,8,22-25]). Five of these traits in safflower and sunflower were bridged by at least one lettuce scaffold (days to flower, disc diameter, number of selfed seed, and achene length and width) and an additional two traits had QTL sharing a homologous gene sequence in all three species (number of heads and achene weight). As expected based on the history of genome duplication within the family, we observed a number of duplicated regions between species and, in some cases, these regions contained relevant QTL. Four QTL corresponding to four traits (achene weight, number of selfed seed, number of heads, and days to flower) in safflower colocalized with multiple QTL in sunflower (i.e., a one-to-several relationship existed for these traits in safflower vs. sunflower QTL comparisons; Additional file 6; [7,8,24]). Interestingly, we identified colocalized QTL for days to flower, achene length, and number of selfed seeds in which the cultivar allele conferred phenotypic effects in the same direction in both species (Figures 2 and 3, Additional file 6), suggesting that the parallel domestication of these traits is the result of parallel selective pressures.

We saw highly significant evidence of QTL colocalization for % oil content (LG I; *P* = 0.001; Figure 3; [23]) as well as marginally significant evidence of QTL colocalization for days to flower (LG I; *P* = 0.08), achene weight (LGs C and H; *P* = 0.07), % linoleic acid (LG H; *P* = 0.08), and achene width (LG C; *P* = 0.09). The significance of the remaining traits that exhibited QTL colocalization between safflower and sunflower was less compelling (*P*-values ranged from 0.12 to 0.21 for disc diameter, number of heads, number of selfed seeds, achene length, and % oleic acid). Nonetheless, when applied across all ten traits with evidence of QTL colocalization, Fisher’s combined probability test was highly significant (*P* = 0.0001) and remained significant even when excluding the highly significant result for % oil content (*P* = 0.004).

## Discussion

### Genetic architecture of safflower domestication

Our results indicate that domestication-related traits in safflower are largely controlled by multiple genes of small to moderate effect. Only two traits (flower color and spininess) had “major” QTL (i.e., PVE > 25%). As such, the genetic architecture of safflower domestication appears similar to that of sunflower, which is the only other crop in which QTL analyses have revealed such a clear paucity of major effect QTL [7,8,24]. More commonly, QTL mapping has suggested that domestication-related traits are conditioned by a relatively small number of loci with large phenotypic effects (reviewed in [63,64]). Recent population genomic analyses have, however, revealed that much larger numbers of genes are typically under selection during crop domestication and/or improvement ([65,66]; reviewed in [67,68]). These findings suggest that the genetic architecture of domestication traits is likely to be complex, even for crops in which initial QTL-based approaches have suggested otherwise.

The single largest QTL identified in our study explained 63.4% of the phenotypic variance in flower color. Further, our observation of 3:1 segregation of flower color suggests that differences in the production of carthamine (the quinochalcone pigment responsible for the production of red florets) within our mapping population are due to the effects of a single locus. Earlier crossing studies of safflower suggested that multiple genes influence flower color [69,70] and more recent studies have shown that there are at least two interacting genes differentiating orange and yellow florets [71]. The fact that we identified just a single QTL suggests that the mapping parents in our population differ primarily in terms of the production of carthamine as opposed to the other floral pigments. More generally, the findings of single, large effect QTL for the presence of a particular floral pigment as well as for leaf spines are consistent with the views of Gottlieb [72], who argued that presence/absence characters and major or structural differences in plants are commonly controlled by just one or two genes.

### Map features and QTL distribution

All but 14 of the markers analyzed exhibited Mendelian segregation ratios, with eleven of these markers occurring within two distorted regions. Though the cause of this distortion remains unknown, it may be due to genomic divergence between the mapping parents in these regions. In this light, it is worth noting the distorted region on LG K harbors QTL for seed-related traits and the distorted region on LG L harbors the large effect QTL for spininess as well as other QTL for internode length, number of internodes, disc diameter, capitulum height, and leaf shape.

In terms of overall marker distribution, we observed numerous tight clusters across multiple LGs. These marker clusters could be a byproduct of an uneven distribution of genes across the genome (recall that all SNPs employed in this study were selected from transcribed sequences), chromosomal rearrangements that differentiate the mapping parents, and suppress recombination in affected regions (though the F_1_ hybrids did not seem to suffer reduced fertility), or – perhaps more likely – the suppression of recombination in and near centromeres. Clustering has also been reported in other genetic maps generated from *C. tinctorius* × *C. tinctorius* crosses, though to a lesser extent [73,74].

We likewise observed a number of QTL clusters across the genome. In some cases, these clusters appeared to co-occur with the aforementioned marker clusters, suggesting that they may be mapping to gene dense regions or to regions with suppressed recombination. It has been argued that species in which the genes underlying domestication-related traits are clustered may be inherently easier to domesticate [75]. In this context, it is worth noting that clustering of domestication-related loci has previously been documented in a number of other crops, including maize [5], common bean [6], pearl millet [76], pepper [77] and sunflower [7] (reviewed in [78]). While Pernès [79] predicted that the linkage of domestication genes can aid cross-pollinated crops in maintaining trait complexes that comprise the domestication syndrome, and further modeling has supported this prediction [80], empirical studies (including the present study) have indicated that these QTL clusters are also found in highly selfing crops [6,81-83]. It is, however, possible that these crops are more allogamous than they seem, or perhaps that increased allogamy occurred earlier in the domestication process, thereby helping to “assemble the domestication syndrome” [75].

### Transgressive segregation

In general terms, transgressive segregation can be produced by complementary gene action, overdominance, and/or epistasis. The former, in which the parents possess alleles with opposing (i.e., antagonistic) effects at complementary genes and thus have the potential to produce segregating progeny carrying an excess of alleles with effects in the same direction [84,85], has been implicated as the most common cause of this phenomenon (summarized in [85,86]). Consistent with this view, the traits with the most extreme transgressive segregation in our population (capitulum height, disc diameter, achene length and width, and achene oil content), were conditioned by multiple QTL (two to seven per trait) and, in many cases, each parental individual carried a mix of alleles with positive and negative effects for these traits. However, we also detected evidence of overdominant QTL effects for three of the traits exhibiting transgressive segregation (total seeds, achene length, achene width, and seed weight). Therefore, neither overdominance nor pseudo-overdominance can be discounted. Finally, many of these same traits were influenced by multiple genetic interactions, suggesting that epistasis could have played a role in producing the observed transgressive segregation – though instances of epistasis were certainly not limited to traits exhibiting transgressive segregation.

### QTL colocalization

The numerous instances of QTL colocalization that we observed suggests that there are many parallels in the genetic architecture of domestication-related traits between safflower and sunflower. While additional work, including fine-mapping, positional cloning, and functional analyses, will be required to establish with certainty that the same underlying genes are responsible for these instances of colocalization, our findings are consistent with the view that selection may, in some cases, have acted in on the same genes during the independent domestications of safflower and sunflower. Such parallel genotypic evolution has been observed in other animal and plant systems, including the evolution of red and green color vision in multiple vertebrate species [87] (reviewed in [88]), the ability of bats, dolphins, and whales to echolocate [89,90] (reviewed in [88]), herbicide resistance in maize and cocklebur [91] (reviewed in [92]), and the glutinous phenotype in rice [93-95], Chinese waxy maize [96], and foxtail millet [97] (reviewed in [64]). Going forward, an improved understanding of the genes underlying parallel trait transitions will provide key insights into the repeatability of evolution, helping us to better predict the phenotypic effects of genotypic changes across a broad array of crops.

## Conclusions

Here, we have presented data demonstrating the complex nature of domestication-related traits in safflower. Our work thus contributes to a growing body of literature showing that crop origins are genetically more complex than once thought. By comparing our QTL mapping results to those from previous studies in sunflower, we have further documented the existence of numerous apparent parallels in the genetic architecture of domestication-related traits within the Compositae. Taken together, these results suggest that selection targeting some of the same genes in safflower and sunflower may have contributed to the parallel trait transitions that occurred during their independent domestications. This work also sets the stage for future analyses aimed at identifying the genes underlying these agronomically important traits, making it possible to further test hypotheses regarding the genetic basis of parallel phenotypic evolution. Such efforts will not only provide critical insights into the repeatability of evolution, but will also facilitate the continued development of safflower as an oilseed crop.

### Availability of supporting data

The data sets supporting the results of the article are available in the NCBI dbEST (*C. tinctorius* ESTs; http://www.ncbi.nlm.nih.gov/dbEST) [GenBank:EL372565-EL412381 and EL511108-EL511145] and will become available in the NCBI SRA (*C. palaestinus* 454 reads; accession number SRR953755) and the Dryad Digital Repository (*C. palaestinus* assembly; http://dx.doi.org/10.5061/ 783 Q4dryad [NNNN]).

## Competing interests

The authors declare that they have no competing interests.

## Authors’ contributions

JMB and SAP conceived the study. SAP produced the mapping population, generated the phenotypic data, designed the genotyping assay, and generated the genotypic data. SR-C-W and RWM were involved in the sequencing and assembly of the lettuce genome. SAP, JMB, and JEB analyzed the safflower data and performed the comparative genomic analyses. SAP and JMB drafted the manuscript with input from the other authors. All authors read and approved the final manuscript.

## Supplementary Material

Additional file 1 **Details regarding library preparation, 454 sequencing, and assembly of the *C. palaestinus *** **transcriptome.** Click here for file

Additional file 2 Summary of sunflower domestication QTL for traits homologous to those investigated in the present safflower QTL study. Click here for file

Additional file 3 Pairwise Spearman correlation coefficients of the 24 traits phenotyped in this study. Click here for file

Additional file 4 Target sequences used for GoldenGate Assay design and their corresponding map positions. Click here for file

Additional file 5 Summary of significant interactions detected among all mapped markers. Click here for file

Additional file 6 **Additional colocalizing quantitative trait loci (QTL), following the format of Figure **3**.** Click here for file
