# Supplementary material for: Genetic analysis of safflower domestication
Source: BMC Plant Biol. 2014 Feb 6;14:43. doi: 10.1186/1471-2229-14-43 (PMC3925122; doi:10.1186/1471-2229-14-43)
Supplement: Additional file 1 — Details regarding library preparation, 454 sequencing, and assembly of the C. palaestinus transcriptome. [file 1471-2229-14-43-S1.docx]

**Additional file 1.** Details regarding library preparation, 454 sequencing, and assembly of the *C. palaestinus* transcriptome.

Library preparation and normalization for 454 sequencing of the *C. palaestinus* transcriptome followed previously described protocols (see text). Briefly, because the 454 technology is unable to accurately sequence strings of homopolymers, a modified “broken chain” oligo-dT primer was used during cDNA synthesis to reduce the length of the mononucleotide runs associated with the 3’ poly-A tail in mRNA. The resultant cDNA was amplified and normalized using the TRIMMER DIRECT cDNA normalization kit (Evrogen, Moscow, Russia). After normalization, the cDNA was sheared into 500-800 base pair fragments and any remaining small fragments were removed using AMPure SPRI beads. The fragmented ends of the remaining cDNA were then polished and ligated to adapters and the desired ligation products were selectively amplified and size selected.

The *C. palaestinus* 454 EST library was sequenced on the GS-FLX at the David H. Murdock Research Institute (http://www.dhmri.org/about.html ) using standard Titanium chemistry (http://www.454.com/). The 454 reads were cleaned using ESTclean (http://sourceforge.net/projects/estclean/) and the cleaned sequences were assembled in MIRA v. 3.0.3 (http://www.chevreux.org/projects_mira.html) using the assembly mode “denovo, est, accurate, 454” and the settings “-AS:mrl=50 -OUT:sssip=yes -CL:bsqc=yes.”
