# Supplementary material for: Genetic analysis of safflower domestication
Source: BMC Plant Biol. 2014 Feb 6;14:43. doi: 10.1186/1471-2229-14-43 (PMC3925122; doi:10.1186/1471-2229-14-43)
Supplement: Additional file 5 — Summary of significant interactions detected among all mapped markers. [file 1471-2229-14-43-S5.docx]

**Additional file 5.** Summary of significant interactions detected among all mapped markers.

| Trait | Type of interaction^ab^ | | |
| --- | --- | --- | --- |
|  | A × A | A × D | D × D |
| Rooting rate |  | I × A | D × I |
|  |  | J × C |  |
| Average leaf roundness | H × K | A × I |  |
|  | H × L | L × L |  |
| Spininess | H × L |  |  |
|  | I × J |  |  |
| Days to flower | A × E |  | A × B |
| Primary capitulum height | E × J | A × K | G × G |
|  | G × G | F × H | J × L |
|  | K × L | G × G | L × K |
|  |  | L × K |  |
| Primary disc diameter | D × J | K × A | B × D |
|  |  |  | D × J |
| Number of heads | A × E | E × G | C × I |
| Flower color | A × D | B × H | B × H |
|  | D × E | I × H | E × I |
|  | D × K |  |  |
|  | E × I |  |  |
|  | L × L |  |  |
| Stem height |  |  | E × H |
| Number of internodes |  | E × E | G × L |
| Internode length | E × K | E × E | B × D |
| Number of selfed seed | B × E | D × D | B × L |
|  | E × L | J × D | C × L |
|  | H × L |  |  |
| Achene weight | A × C | E × K |  |
| Achene length | A × C | A × L | C × K |
|  |  | D × E |  |
|  |  | F × E |  |
|  |  | K × C |  |
| Achene width | A × C | A × I |  |
|  |  | A × L |  |
|  |  | D × J |  |
|  |  | E × K |  |
| Seed viability | A × L | A × L | A × G |
