# Supplementary material for: Genetic analysis of safflower domestication
Source: BMC Plant Biol. 2014 Feb 6;14:43. doi: 10.1186/1471-2229-14-43 (PMC3925122; doi:10.1186/1471-2229-14-43)
Supplement: Additional file 6 — Additional colocalizing quantitative trait loci (QTL), following the format of Figure 3. [file 1471-2229-14-43-S6.pdf]

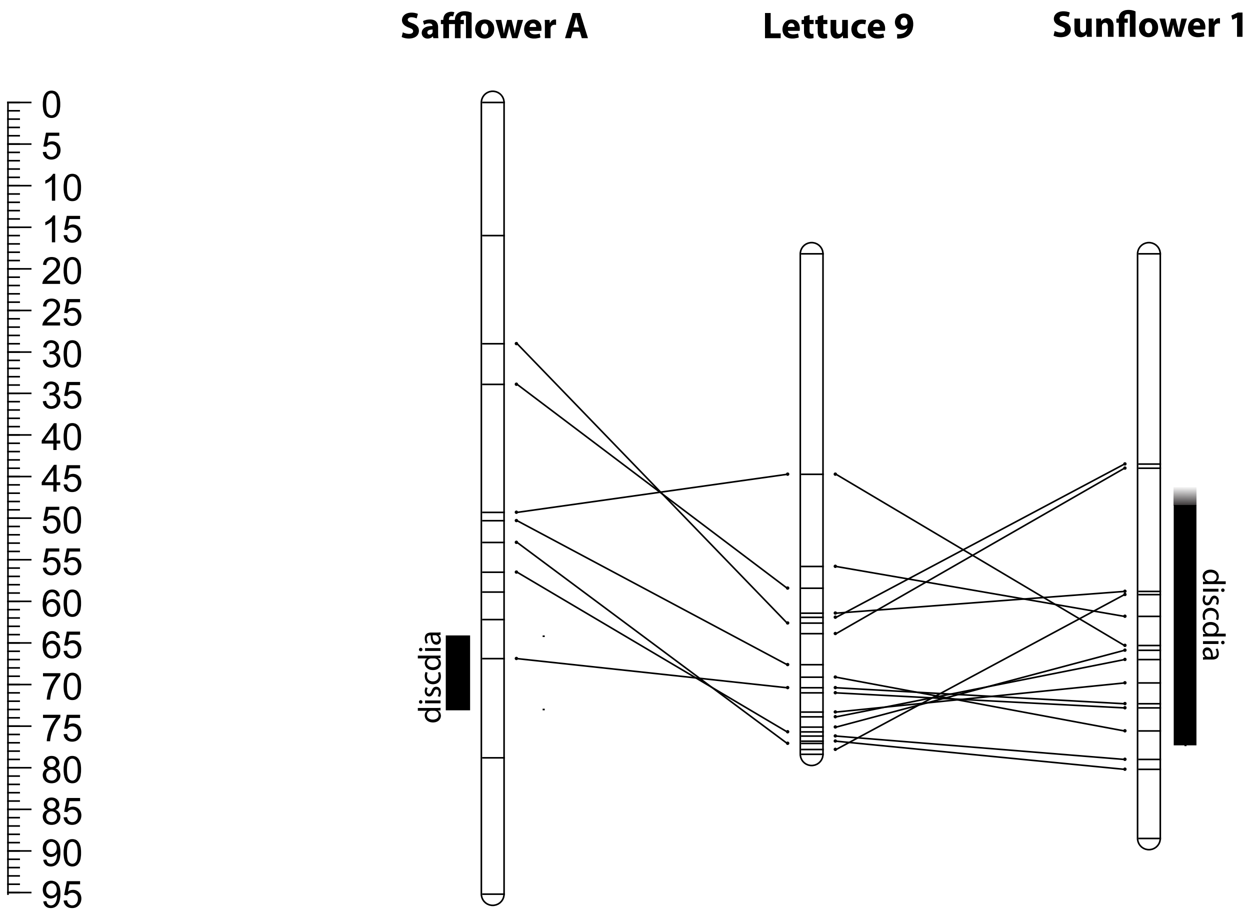
Safflower A
Lettuce 9
Sunflower 1
0
5
10
15
20
25
30
35
40
45
50
55
60
65
70
75
80
85
90
95
discdia
discdia

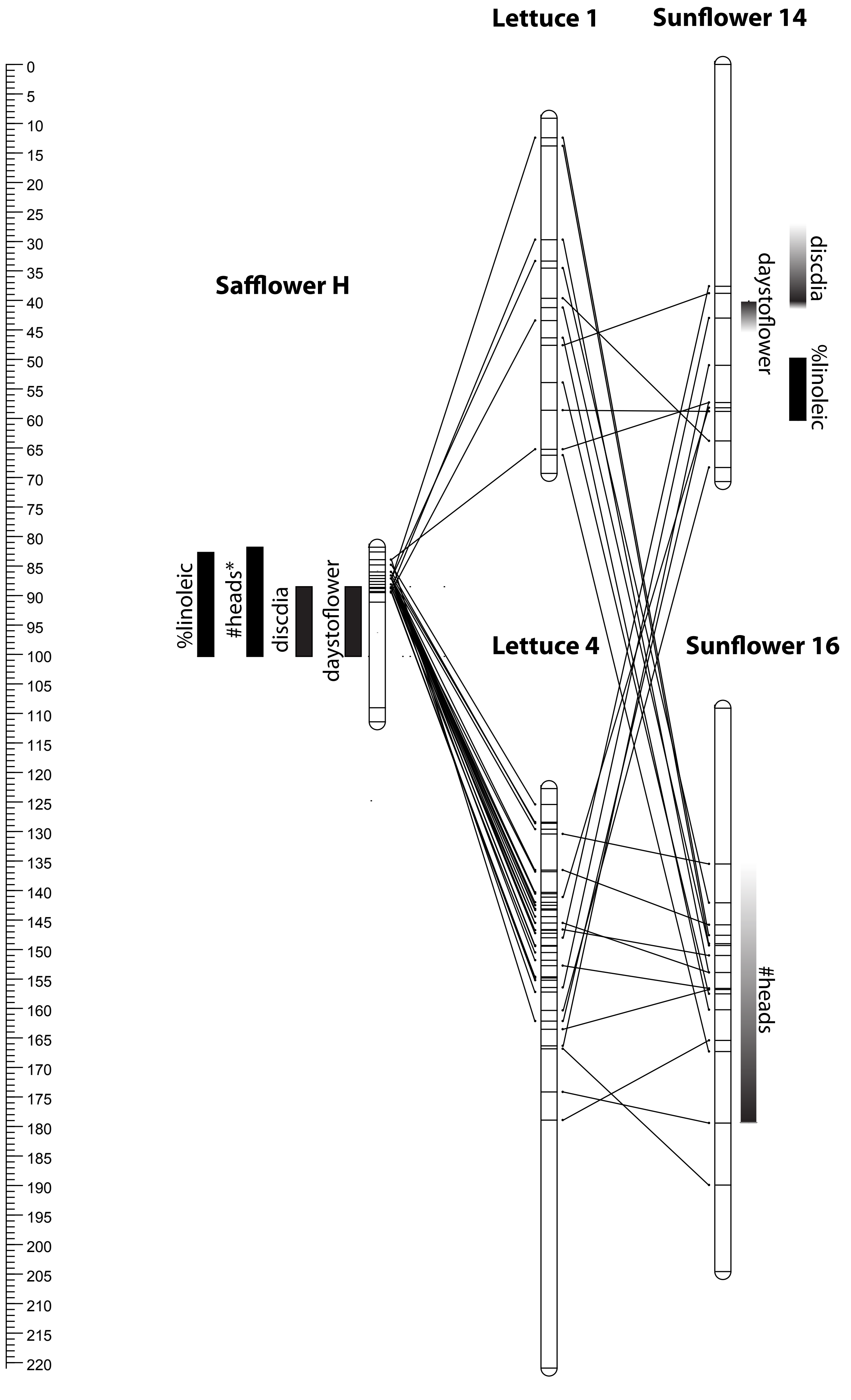

Lettuce 1
Sunflower 14
Safflower H
Lettuce 4
Sunflower 16
%linoleic
#heads*
discdia
daystoflower
discdia
daystoflower
%linoleic
#heads
0
5
10
15
20
25
30
35
40
45
50
55
60
65
70
75
80
85
90
95
100
105
110
115
120
125
130
135
140
145
150
155
160
165
170
175
180
185
190
195
200
205
210
215
220

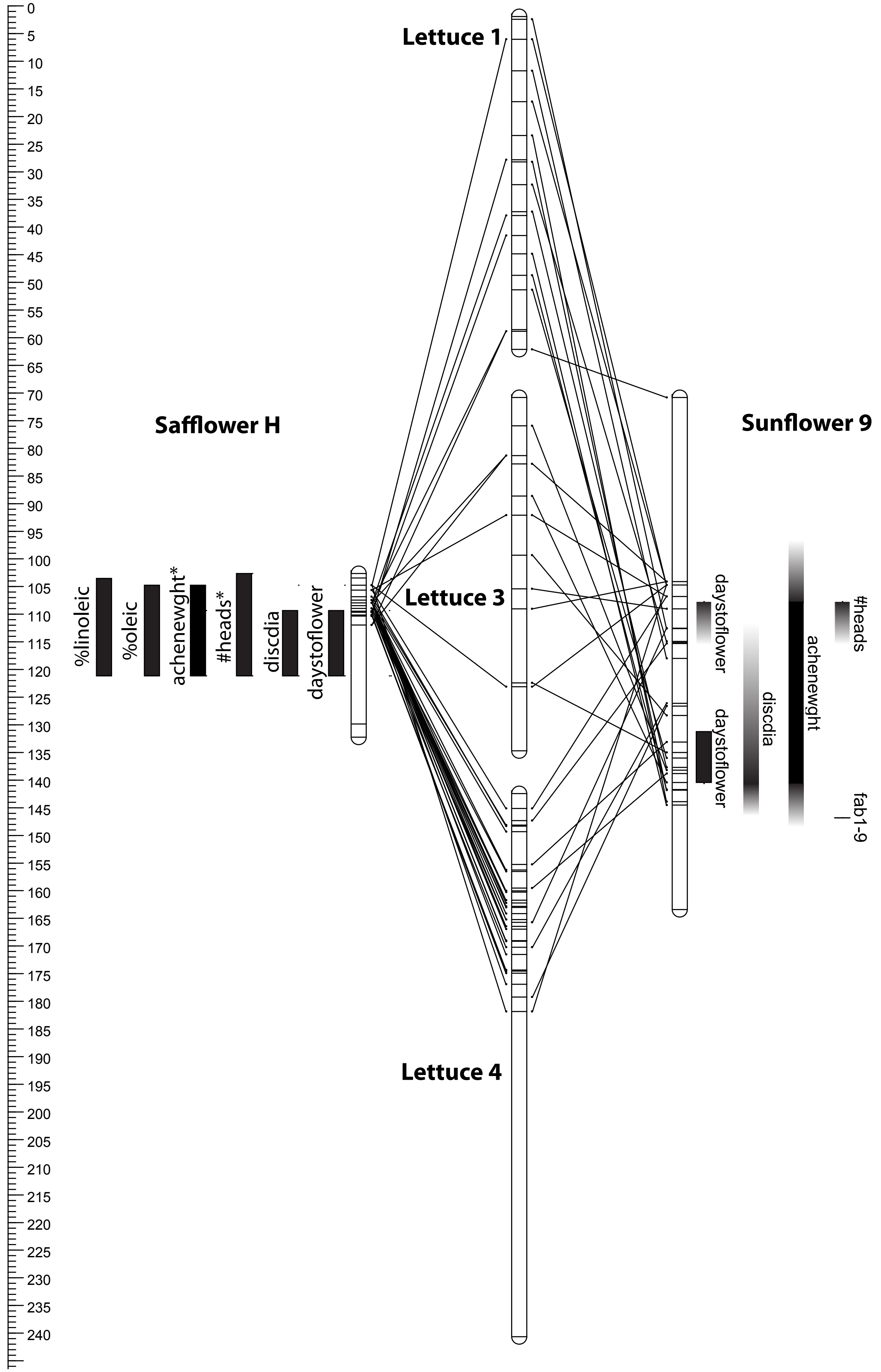

Lettuce 1
Safflower H
Sunflower 9
Lettuce 3
Lettuce 4
%linoleic
%oleic
achenewght*
#heads*
discdia
daystoflower
daystoflower
discdia
achenewght
#heads
daystoflower
fab1-9
0
5
10
15
20
25
30
35
40
45
50
55
60
65
70
75
80
85
90
95
100
105
110
115
120
125
130
135
140
145
150
155
160
165
170
175
180
185
190
195
200
205
210
215
220
225
230
235
240

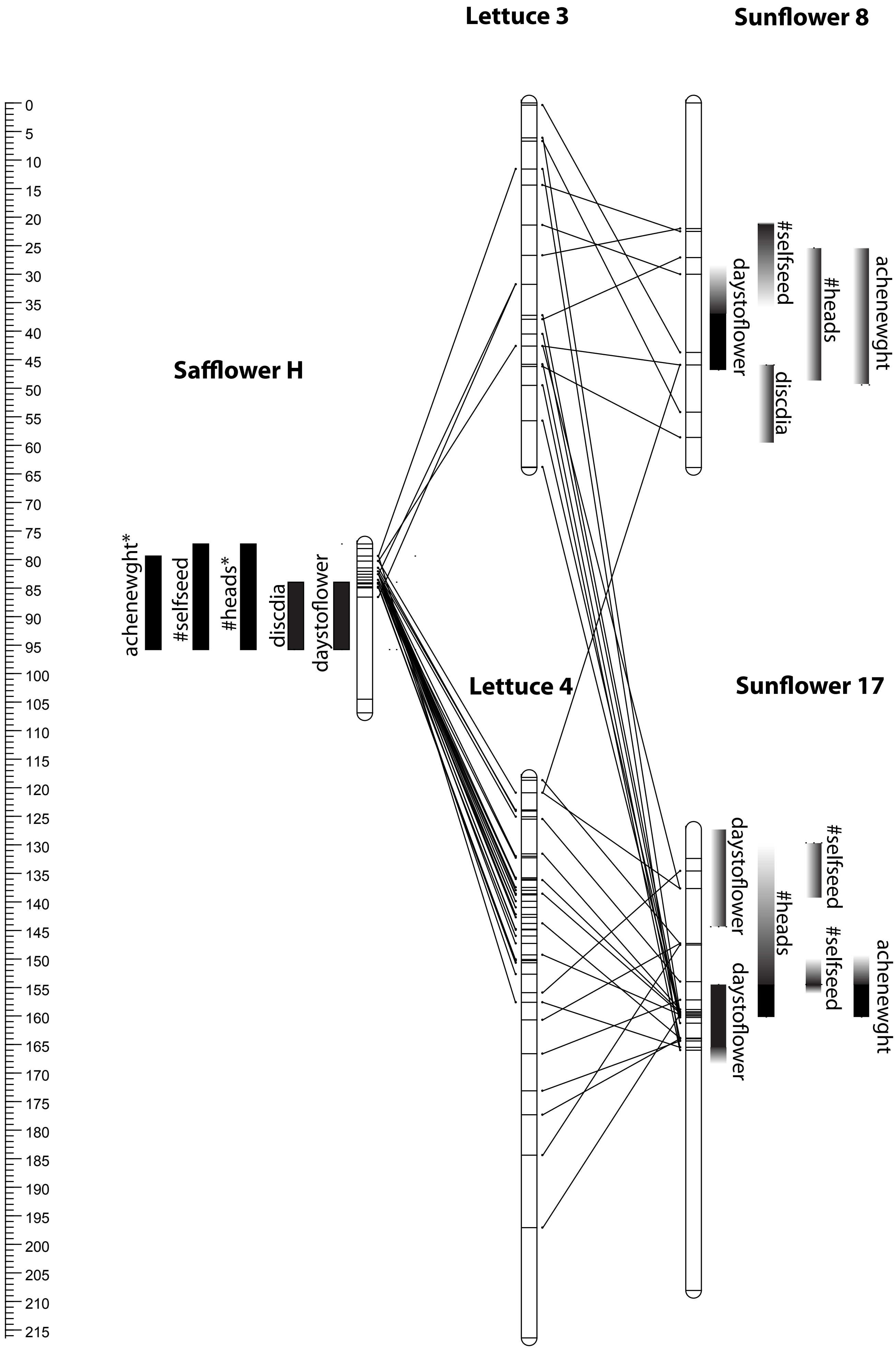

Lettuce 3
Sunflower 8
Safflower H
Lettuce 4
Sunflower 17
achenewght*
#selfseed
#heads*
discdia
daystoflower
#selfseed
daystoflower
#heads
achenewght
discdia
daystoflower
#selfseed
#heads
#selfseed
achenewght
daystoflower
0
5
10
15
20
25
30
35
40
45
50
55
60
65
70
75
80
85
90
95
100
105
110
115
120
125
130
135
140
145
150
155
160
165
170
175
180
185
190
195
200
205
210
215

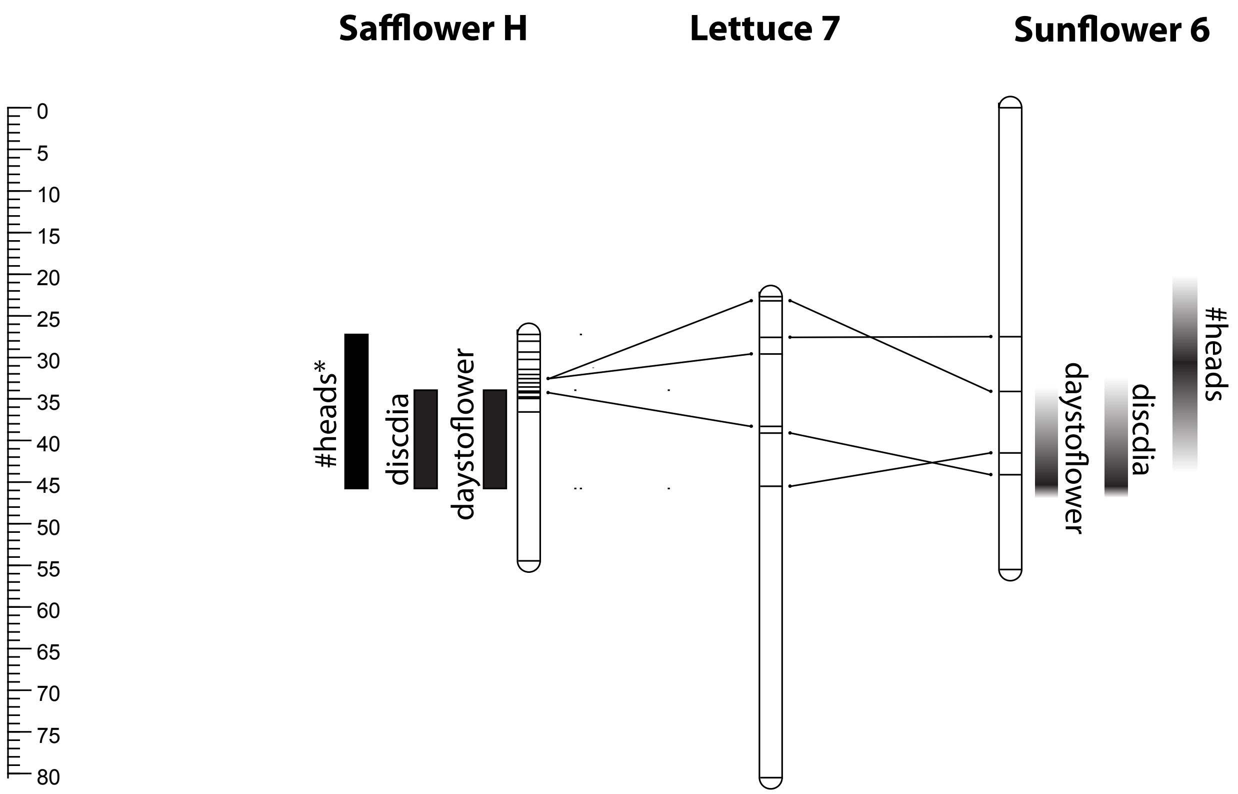
Safflower H
Lettuce 7
Sunflower 6
#heads*
discdia
daystoflower
daystoflower
discdia
#heads

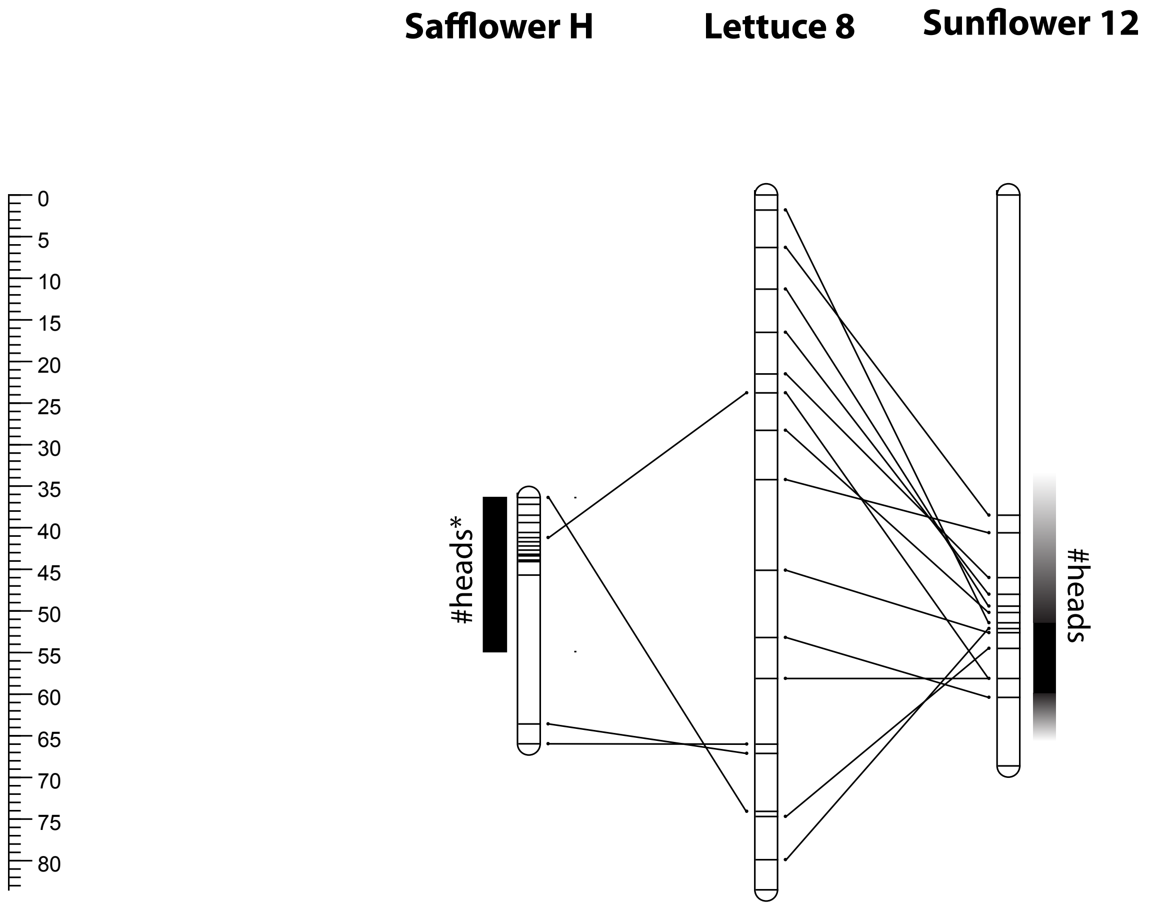
Safflower H
Lettuce 8
Sunflower 12
#heads*
#heads
